# Decoupled, wearable soft robotic rehabilitation device for the upper limb

**DOI:** 10.1017/wtc.2025.10018

**Published:** 2025-08-07

**Authors:** James Greig, Mhairi McInnes, Edward K. Chadwick, Maria Elena Giannaccini

**Affiliations:** 1 School of Engineering, University of Aberdeen, Aberdeen, UK; 2 School of Computer Science, University of Nottingham, Nottingham, UK

**Keywords:** biomechanics, design, exosuits, rehabilitation robotics, soft wearable robotics

## Abstract

Lightweight, adjustable, and affordable devices are needed to enable the next generation of effective, wearable adjuncts for rehabilitation. Used at home or in a rehabilitation setting, these devices have the potential to reduce compound pressures on hospitals and social care systems. Despite recent developments in soft wearable robots, many of these devices restrict the range of motion and lack quantitative assessment of moment transfer to the wearer. The decoupled design of our wearable device for upper-limb rehabilitation successfully delivers almost the full range of motion to the user, with a mean maximum flexion angle of 149° (SD = 8.5). In this article, for the first time, we show that in tests involving a wide range of participants, 82% of the moment produced by the actuator is applied to the wearer. This testing of elbow flexion moment transfer supports the effectiveness of the device. This research is a step toward effective pneumatic soft robotic wearable devices that are adaptable to a wide range of users – a necessary prerequisite for their widespread adoption in health care.

## 1. Introduction

Soft robotic wearable devices offer great potential for assisting individuals with muscle weakness in the performance of activities of daily living (ADL), support exercise in rehabilitation for people following stroke or spinal cord injury, and even potentially providing assistance with work-related tasks for able-bodied users (Bardi et al., 2022). In the case of rehabilitation, which is the focus of our work, there is a clear benefit to be derived from increasing the amount of therapy offered by supplementing therapist time with robotic assistance. One in four adults over 25 years will have a stroke in their lifetime (WSO, 2023), and there are currently approximately one hundred thousand strokes per year occurring in the United Kingdom (SA, 2022). The effects of a stroke can be wide-ranging, and debilitating upper-limb impairment occurs in 50–80% of acute cases and remains in 40–50% of chronic cases (Hussain et al., 2018). Although rehabilitation plays a large role in the overall recovery of a patient, the Chartered Society of Physiotherapy recognizes that there is a significant unmet need for rehabilitation (CSP, 2024). In spinal cord injury, high-level injury leads to upper-limb, paralysis where recovery will be limited.

The 2023 UK National Clinical Guideline for Stroke (NCGS 2023) recommends robotic rehabilitation as a potential adjunct for usual therapy. Research into the use of robotic devices for upper-limb rehabilitation has been ongoing since the early 1990s, with the work done on the MIT MANUS forming some of the seminal literature in the field (Hogan et al., 1992). Despite their potential, robotic rehabilitation devices are still uncommon in both clinical and home settings. Laparidou et al. (2021) conducted a systematic review of the perceptions of device users, carers, and therapy staff who had been involved in robotic rehabilitation. It highlights the appeal of robotic rehabilitation – allowing users to take more control over their recovery and the ability to provide quantitative feedback to patients and therapists, improving motivation and monitoring effectiveness as well as increasing the rehabilitation volume. The authors also summarized the main barriers to widespread implementation: the accessibility, cost, and time to set up conventional robotic devices, those built using motors and rigid linkages, as seen in the majority of robots. Soft robotic devices can be built with low-cost materials, making them potentially much more accessible to a wide range of users.

Soft robotics is based on the principle of compliance, and there are two common approaches to this goal (Whitesides, 2018). The first is to build robots from rigid materials and use torque sensing and impedance control to allow collaboration and contact with humans without causing harm. The approach we have chosen is the use of soft materials to build the robotic actuators, which are designed to be simple, light, and low cost. In addition, the flexibility of the soft robotic actuator is an intrinsic safety feature, as it does not need to be perfectly aligned to the human skeleton and joints as a rigid exoskeleton does. This reduces the occurrence of misalignment that creates undesired interaction forces that can decrease comfort and safety.

In this article, we explore the design of a wearable soft robotic device, which is intended to be used in assisting early stage rehabilitation of the upper limb, where assistance in elbow flexion is required. We use a biomechanical simulation to derive device requirements and present solutions to common issues, such as limited range of motion, and deriving meaningful, quantitative information about their performance both on the bench and on the human. In the next section, our device is compared with the state-of-the-art, followed by biomechanical modeling in Section 3, the development of the device in Section 4, and the human study in Section 5 that tests the effectiveness of the device and for which we have selected a wide range of user age and sizes to investigate adaptability in different users.

## 2. Background

**Figure 1.**
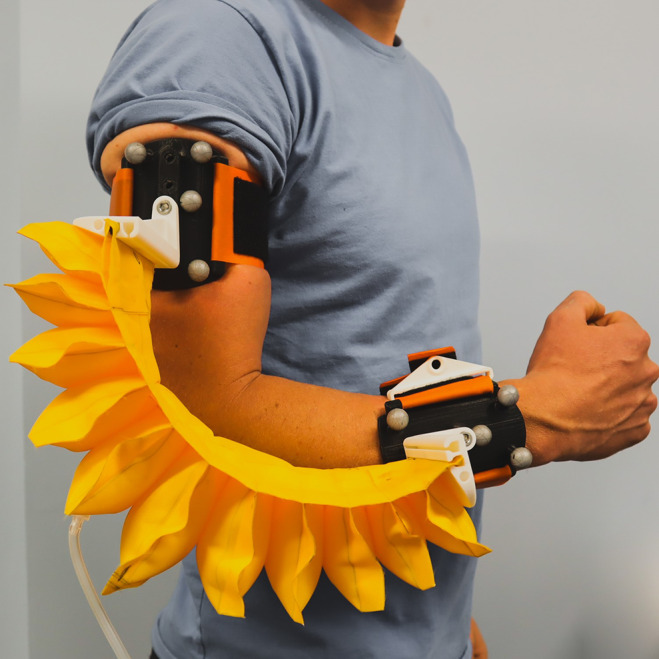
Wearable soft robotic device attached to the upper and forearm, with the thermoplastic polyurethane (TPU) pneumatic actuator attached alongside the wearer’s arm. Bending occurs in a plane that is perpendicular to the elbow flexion axis and incorporates the two attachments shown in black. The wearable device is “decoupled” from the arm, meaning that it is only attached to the arm in two points – the forearm and upper arm cuffs. This allows the actuator to bend at an angle that is different from the elbow flexion angle.

The selection of the pneumatic bending actuator shown in Figure 1 followed an extensive assessment of existing devices and their limitations. (Bardi et al., 2022) reviewed soft robotic devices for the upper limb, which have been developed for rehabilitation and assistance for healthy individuals. Many devices use linear actuators, such as motor-driven cables (Chiaradia et al., 2018) and McKibben pneumatic actuators (Belforte, 2018), as well as more recent woven actuators, including those made with active textiles (Hiramitsu et al., 2019). While this type of actuator can produce high tensile forces, its main challenge is in the creation of sufficient moment arms to generate the required moment about the joint. In the elbow, this occurs at low angles of flexion and can result in large shear forces between the user and the cable attachment (Chiaradia et al., 2018; Harbauer et al., 2020).

Pneumatic bending actuators produce a bending motion upon pressurization. They can be made with woven actuators, such as in Tschiersky et al. (2020), where thin McKibben muscles in combination with a flexure strip are used, or (Xiang et al., 2018), where McKibben muscle and SMA-fishing line are utilized. More recently, pneumatic soft bending actuators have been made with textiles, such as those described by Koh et al. (2017), (Nassour et al. (2021), and Thalman et al. (2018). A major benefit of these bending actuators is that the tip force of the actuator can be near-perpendicular to the axis of the body segment that it is attached to, providing much larger moment arms that generate the desired motion more safely and efficiently. These devices use TPU-based actuators for flexion, by either using TPU films within sewn nylon pockets (Thalman et al., 2018) or by using reinforced TPU-coated Nylon fabrics within a separate textile structure (Nassour et al., 2021; Dávila-Vilchis et al., 2022). The benefits of TPU films over a molded elastomeric design, as presented in Koh et al. (2017), include higher working pressures and simpler construction, although the main advantage is the low mass and low volume when the actuator is deflated. This allows a device to be lightweight and flexible, which is important for adaptability and ease of use. To reduce the complexity of existing devices that utilize either multiple separate air-holding chambers (Thalman et al., 2018) or multiple materials (Thalman et al., 2018; Nassour et al., 2021; Dávila-Vilchis et al., 2022), we have developed a single-chambered, single-material TPU soft actuator.

Achieving the full range of motion of the joint is necessary for effective rehabilitation, particularly in reducing the effects of spasticity (Stevenson, 2010). As a minimum, the range of motion should meet the requirements for typical ADLs, as restoring the ability to perform ADLs is one of the main objectives of rehabilitation. When performing typical ADLs, individuals spend most of their time in the range of 40–120 degrees of elbow flexion (Haverstock et al., 2020). However, some critical ADL tasks, such as eating, drinking, and washing, require the full range of elbow flexion, which is typically 150° (Rosen et al., 2005; An and Morrey, 2018).

As the creation of soft pneumatic bending actuators with sufficient curvature to meet this 150° range of motion is relatively simple, the challenge lies in the connection of these actuators to the limb. All devices for assisting elbow flexion so far have placed the actuators behind the elbow and continuously connected them to the upper arm and forearm (Koh et al., 2017; Thalman et al., 2018; Nassour et al., 2021; Dávila-Vilchis et al., 2022). This seems intuitive, as the pressure exerted at the ends of the actuator can be transmitted directly to the limb, and in this position, the actuator is unlikely to get in the way of the user. However, attaching the actuator to the arm along its full length forces it to conform to the angles of the limb, which may not match the actuator’s natural bending behavior. The textiles required for this continuous connection along the length of the actuator can also restrict movement. Second, as the olecranon process (the bony tip of the elbow) extends beyond the axis of rotation of the elbow joint, the length of the actuator must increase as the elbow flexes.

Continuously connecting the wearable device to the upper arm and forearm results in either a restriction in the range of motion, with the best of existing wearable devices achieving only 118° (Koh et al., 2017), or a requirement for elastic connections to the limb, which can lead to poor moment transfer (Proietti et al., 2022). Our solution to increasing the range of motion is a decoupled design, whereby the actuator is connected to the upper limb at its ends only, as shown in Figure 1, meaning the actuator is bending continuously and does not mirror the sharp bending at the biological elbow. Only the ends of the actuator are connected to the limb, meaning this is where the moment transfer occurs.

A common measure of the effectiveness of a wearable device is the reduction in surface electromyography (sEMG) through its use. For this reason, in Section 5, we use sEMG to test the ability of our device to help perform a movement and hence its effectiveness.

The leap from bench testing of the actuator to measuring sEMG of a wearer does not give a full picture of the effectiveness of the device, as the attachment between the actuator and the wearer through soft tissue inevitably leads to some moment losses, which must be quantified. A recent study (McCann et al., 2023) on a soft wearable device for the shoulder compared the moment produced when attached to a bench test, a mannequin, and a human. This described significant differences between the moments measured between these tests due to anatomical simplifications of the mannequin, for example, the ability to effectively transfer forces through soft tissue. The torque measured on the human was between 38% and 50% of that on the mannequin, which was also 45% of the torque measured in bench testing, demonstrating significant losses. A thorough understanding of the forces applied to the body is necessary as the first step in assessing the safety of a device, which is an inherent challenge for wearable rehabilitation robots (Bessler et al., 2021). In general, soft tissue-related and musculoskeletal adverse events are regarded to be mainly attributable to forces exceeding safe limits (Bessler et al., 2021). However, no study quantitatively reports the moment transfer from the soft robotic wearable device to the user in the elbow, a gap targeted by this work.

### 2.1. Contribution to the state of the art

In this article, for the first time, the transfer of the flexion moment from the device to the elbow was quantified, showing that 82% of the moment produced by the soft actuator is applied to the wearer. This is shown in the fixed-angle testing experiments (Section 5.2.2).

In addition, the “decoupled” design described in this article demonstrates the ability to assist elbow flexion through almost the full 150° range of motion (Section 5.2.4) as it achieves a mean maximum flexion angle of 149° (SD = 8.5) and a mean range of motion of 132 (SD = 13), compared to existing devices (Koh et al., 2017; Dávila-Vilchis et al., 2022) that have a maximum range of motion of 118°. The novel “decoupled” method of attachment to the arm connects the actuator to the arm only at its ends (see Figure 1). Because of this construction, the actuator bending is separated and decoupled from the elbow joint position, inducing a flexion moment without the need to conform to the shape of the arm, which would decrease its range of motion.

The comprehensive bench testing of our device allows comparison with the results of our biomechanical simulation in OpenSim, described in the next section.

## 3. Biomechanical modeling

### 3.1. Aim

The theoretical moment that needs to be supplied by the rehabilitation device was quantified throughout the whole flexion movement. For a prescribed elbow flexion motion, the additional moment needed was computed using a biomechanical model, which estimated the moment deficit that the user is unable to deliver. The tool used in this study was OpenSim (SimTK), which calculated the necessary assistance required to produce elbow flexion for varying levels of simulated muscle weakness.

### 3.2. Method

The study used the MoBL-ARMS Upper Extremity Dynamic Model (SimTK, 2021) as this provides the necessary degrees of freedom and contains all the major muscles contributing to elbow flexion. Shoulder elevation, rotation, and abduction were locked, as were forearm pronation/supination and deviation/flexion of the wrist, leaving elbow flexion/extension as the single degree of freedom.

Inverse dynamics analysis is used to calculate the net joint moments associated with predefined kinematics, such as elbow flexion, taking account of gravitational, inertial, and contact forces. Static optimization is then used to estimate the muscle forces required to match those net joint moments. In some cases, muscle forces are insufficient to create the required moments, for example, in cases of muscle weakness. In these cases, a hypothetical “reserve actuator moment” is applied to the relevant degree of freedom to achieve a dynamically consistent solution. In our simulations, we equate this value to the additional moment that would be required from an assistive device to complete the movement.

The model was modified such that the upper arm was held in its default natural posture and the elbow joint rotated from fully extended to 130° of flexion. This falls short of the full 150° flexion of the joint (An and Morrey, 2018), a limitation of using the MoBL-ARMs model in this study. Peak elbow moment typically occurs at 90° of elbow flexion (when the upper arm is held against the torso in the coronal plane), as this is where the moment arm of the gravitational force is greatest. This means that the limitation of flexion angle should not affect peak moment calculation, which will be used to determine the suitability of the actuators.

The OpenSim model was modified directly to reduce the “max isometric force” value of each muscle, which is the maximum tensile force produced by the muscle actuator. The model is based on the muscle forces of a 50th percentile adult male, and three files were created to represent models with 2%, 5%, and 10% of the original muscle force. These percentages were chosen from a preliminary study, where at 2%, the reserve actuator moment matched values for elbow flexion moment in the literature (Xiloyannis et al., 2017) and at 10%, the threshold where reserve actuators ceased to provide a significant input. In a study of 19 patients who had experienced a stroke within 6 months, dynamometry was performed giving a pre-therapy range of 1.19–7.02 N in elbow flexion (Kiper et al., 2021). When compared to the mean value for healthy males (as the OpenSim model is based on an average adult male), which gives a maximum elbow flexion moment of 50.9 Nm (Harbo et al., 2012) and applying the moment arm used in the MoBL-ARMs model of 0.33 m, this gives a post-stroke range of 2.3%–13.7% of the mean healthy value. This shows that the chosen range of 2%–10% of the original muscle force is representative of the strength measured in stroke survivors.

**Figure 2.**
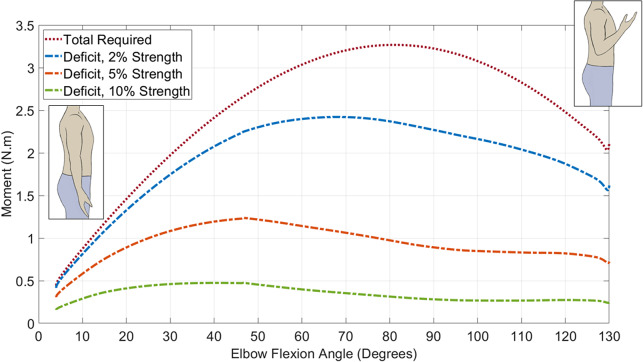
Result of the OpenSim biomechanical simulation of the reserve actuator moment versus elbow flexion angle for each condition. This shows the total moment required to perform the movement (muscle moments + reserve actuator) and the moment deficit for each of the three modeling conditions.

With the modified models, the static optimization tool was used to conduct inverse dynamics, to determine the individual muscle forces required to complete the movement. OpenSim “reserve actuators,” as defined above, are used to compensate for any deficit within the muscle forces and allow it to create a feasible solution. By plotting these, the moment deficit through the whole movement can be quantified. This represents the assistive moment required from the actuator of the wearable device. Figure 2 shows the reserve actuator moments through the range of motion for 2%, 5%, and 10% of the original muscle force.

The results were checked by multiplying the force of each individual muscle by its elbow flexion moment arm (contained within the MoBL-ARMS model). The sum of these moments plus the reserve actuator moment represents the full elbow flexion moment. For each simulation, the total elbow flexion moments were equal, and occurred at 90° of elbow flexion.

### 3.3. Results

Figure 2 shows the reserve actuator moment required at the elbow joint of the model, as the elbow moves from fully extended to 130° flexion. Each trend line shows the three different modeling conditions, where the muscles had either 2%, 5%, or 10% of the original model’s capability, plus the total moment required to complete the movement (including muscle forces). All conditions show a trend that increases from close to zero at the fully extended angle, increasing to a peak, and decreasing once more to a nonzero value at max flexion, as a positive moment is required to keep the arm bent. The reserve actuator moment is greater for low-strength conditions, and the angle at which the peak reserve actuator moment occurs varies for the different load conditions. This is due to the varying contribution of the different muscles, whose moment arms vary with the elbow flexion angle. For an individual with muscle strength equal to 2% of that of a 50th percentile healthy male, the assistance required to complete the prescribed task has a peak of 2.4 Nm. For an individual with 5% of this strength, the peak is 1.2 Nm, and for 10%, 0.5 Nm. The comparison between this model data and the output of our wearable device is described in Section 4.3.

### 3.4. Specification

As the intended use of this device is early stage rehabilitation of individuals with significant paresis, the primary goal is for the actuator to meet or exceed the moment profile specified by the model with 2% muscle strength. This should be demonstrated throughout the full range of elbow flexion.

## 4. Development of device

The device consists of a novel pneumatic soft actuator, plus two attachments to the upper limb, as seen in Figure 1. The design of the device ensures that it does not affect shoulder movement. When deflated, the impedance to elbow flexion and extension is very low, due to the low stiffness of the fabric actuator. Pronation and supination remain possible with the device fitted.

### 4.1. Actuator construction

**Figure 3.**
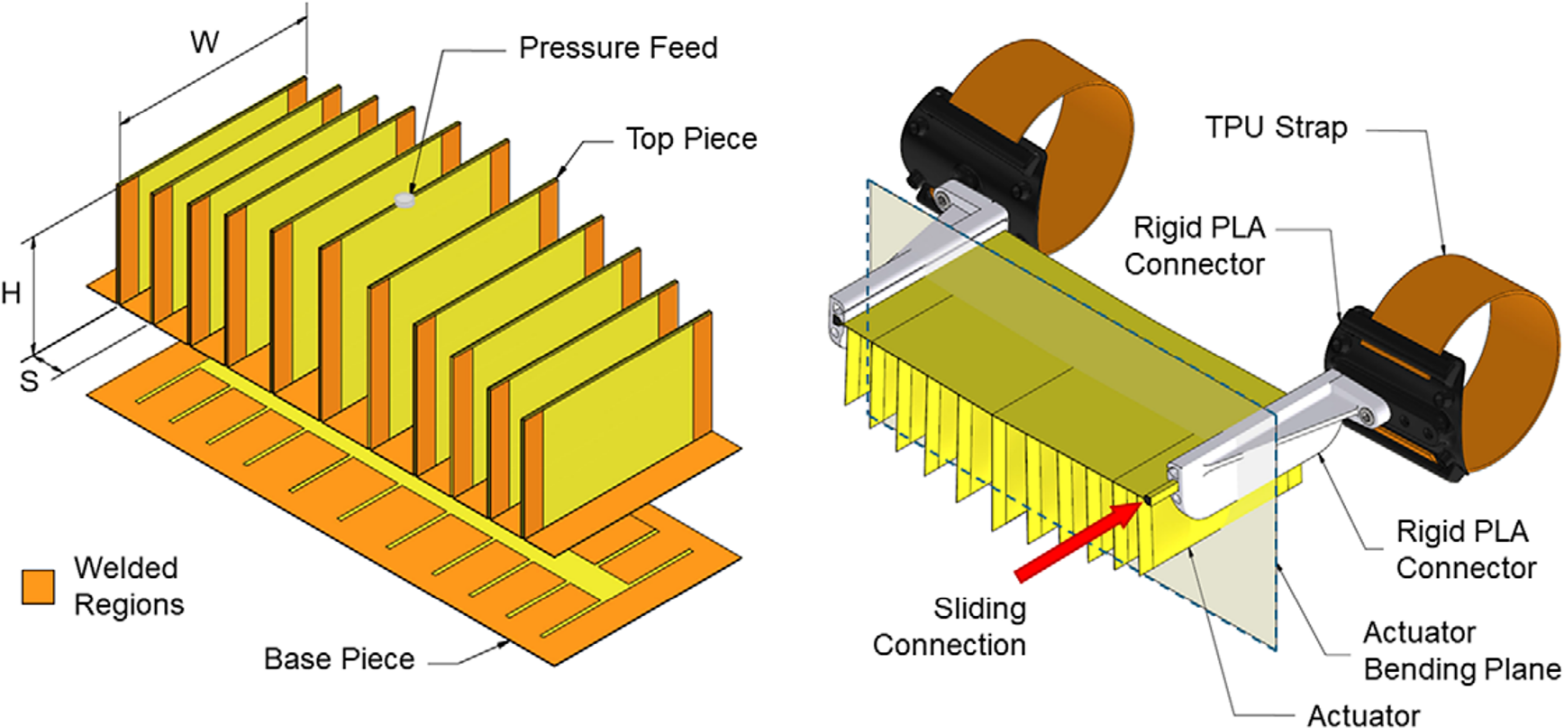
Construction of the TPU actuator (left) and assembly of the device (right). Actuator parameters such as the height (H), width (W), and spacing (S) of the individual chambers can be modified. The actuator bending plane is aligned perpendicular to the elbow axis.

The flexion actuator was constructed from Riverseal 200 (Rivertex Technical Fabrics), a 270 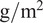 (TPU)-coated nylon, which is heat-weldable at 190  C. TPU was selected due to its high strength and ease of creating pressure-holding seams (Nassour et al., 2021; Dávila-Vilchis et al., 2022). The actuator was made from two pieces – a base piece and a top piece, folded to create individual chambers, as shown in Figure 3. By welding each individual chamber on three sides, a network of chambers can be inflated from a single pressure feed. The use of a single-chambered, single-material TPU actuator is an improvement on existing TPU actuator designs, which typically use a TPU film inside a separate sewn fabric reinforcement (Thalman et al., 2018; Nassour et al., 2021; Dávila-Vilchis et al., 2022) and multichambered designs, which require a separate pressure feed to each chamber (Thalman et al., 2018). Removing the need for a sewn structure and multiple inflation points can simplify the manufacture, supporting our target of a low-cost device.

**Figure 4.**
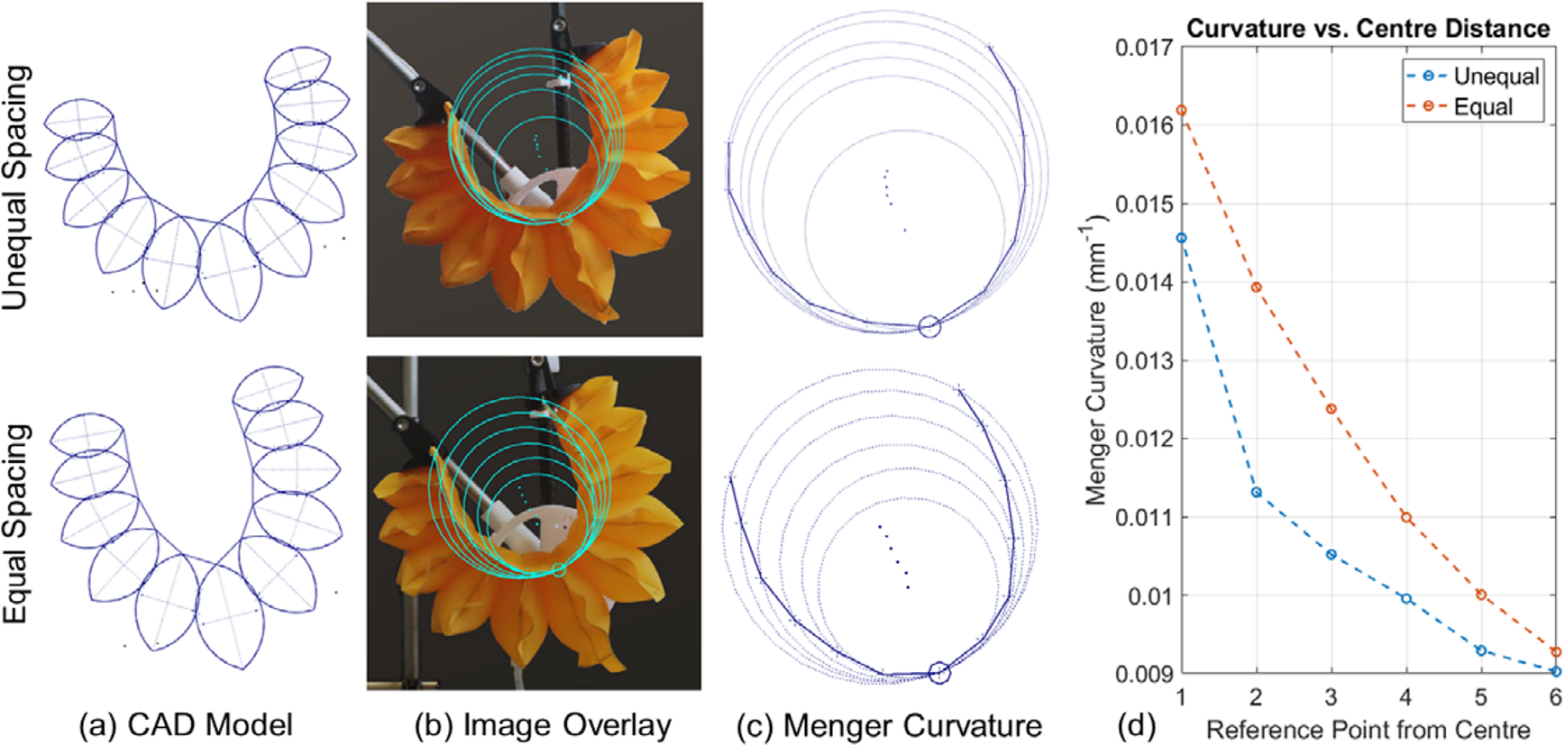
Comparison of the equally spaced and unequally spaced actuators. The CAD model (a) shows curvature prediction based on geometry, while the image overlay (b) shows the method of plotting reference points, with the Menger curvature Radii (c), leading to the plot of Menger curvature (d) for increasing distances from the centerline of the actuator for both equally and unequally spaced actuators.

### 4.2. Actuator geometry

The design parameters of the flexion actuator are manifold, as seen in Figure 3. The width (W), height (H), and spacing (S) of the individual chambers can be changed between designs, as well as the overall length of the actuator. To better understand how these parameters affect the behavior of the actuator, a two-dimensional geometric model was created within Autodesk Inventor, as shown in Figure 4(a). The model was parametrically designed such that the chambers would reduce in height as their width increased. The interaction between chambers was constrained, simulating the contact between them as they inflate, which leads to the overall curvature of the actuator. A constant arc length of the chamber represented the non-extending behavior of the TPU fabric. This method builds a more comprehensive model of the bending analysis when compared to that used in Thalman et al. (2018), which requires assumptions such as a point contact between chambers (where our model allows for adjustment of the contact area) and analysis only of the circular cross section of the fully inflated chamber (where we can model any state from fully deflated to a fully inflated chamber).

**Figure 5.**
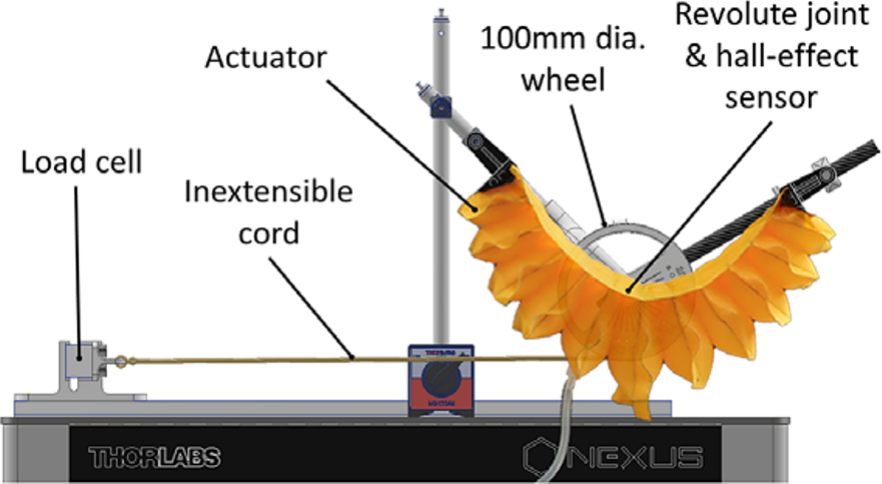
Rig used for bench testing the actuator curvature and moment. A wheel at the axis of a revolute joint mimics the human elbow. An inextensible cord connecting the circumference of this wheel to a load cell allows torque measurement.

In order to test the difference in bending behavior and moment production caused by design variation, two actuators were modeled and built, identical in every parameter except for the spacing between the chambers (S), with a chamber height (H) between 15 and 75 mm and a width (W) of 100 mm. One actuator was built with equal spacing, and the other was built with an equal ratio of chamber height and spacing, resulting in larger spacing at the center and gradually smaller spacing toward the ends, which we call an unequally spaced actuator. Both actuators are shown in Figure 4(b).

A test fixture was built to simulate an elbow, with a revolute joint that can be fixed at angles ranging from 0° to 150°, in 10° increments, as shown in Figure 5. A rotary hall-effect sensor (PSC-360G2, Piher Sensing Systems) recorded the flexion angle, and a differential pressure sensor (SSCSNBN030PDAC5, Honeywell) monitored the feed pressure to the actuator. The pressure was supplied by a 6 L compressor, which fed air at 2 bar to a pneumatic regulator (ITV1030-01F2N3, SMC), which provided a pressure of 0.5 bar to the actuator. MATLAB was used via a DAQ (NI-USB-6341, National Instruments) to control the signal to the regulator and read the output of the pressure and rotary sensors.

The actuators were attached to the test rig and constrained at the desired elbow angle. They were inflated to 0.5 bar and an image was captured, then imported into Autodesk Inventor, scaled, and the reference points recorded. By plotting a circle that lies on three reference points, a Menger Curvature can be calculated as 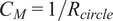 . This was then plotted for a series of points, as shown in Figure 4(d). This testing was repeated for both the equally spaced and unequally spaced actuators.

Figure 4 shows the Autodesk model, sample photograph, and Menger curvature for both the equally and unequally spaced actuators. These demonstrate the ability to modify the curvature along the length of the actuator for the desired application. In this case, the equally spaced actuator has a much higher Menger curvature at the center of the actuator when compared to the unequally spaced actuator, with values of 0.0162 and 0.0145 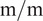 , respectively (see Figure 4(d)). As the centerline of the actuator is aligned with the elbow joint axis, the Menger curvature of the actuator is identical to the Menger curvature of the elbow joint. The overall behavior of the equally spaced actuator created a sharper bending angle, as seen in Figure 4(a) and (c). The difference between these results is proof of the embodied intelligence paradigm, which states that morphology influences behavior. However, both actuators were able to achieve the 150° bending angle required to completely flex the elbow.

### 4.3. Actuator moment

To determine the moment produced by the actuators, the aforementioned revolute joint test rig was used, with the addition of a 20 kg load cell (OBUG-20kg-0.4-000, Applied Measurements). A 100 mm diameter wheel attached to the pivot point of the test rig was connected to the load cell via an inextensible cord to measure moment at the revolute joint at angles of 0°–150° at increments of 10°. To determine the maximum safe working pressure of the actuator, burst tests were carried out on sample chambers, which showed an average burst pressure of 3.0 bar. Applying a safety factor of 2 to this gave a maximum safe working pressure of 1.5 bar.

The actuator moment versus elbow angle for both the equal and unequally spaced actuators was initially tested at 0.5 bar. It was shown that both actuators produced similar moments, which were highest at the low angles of flexion and gradually reducing as the bending angle of the test rig increased. At angles below 45°, the unequally spaced actuator produced a higher moment and a higher peak overall moment. However, at angles over 45°, the equally spaced actuator produced a consistently higher moment, with a difference of around 5%. At 90°, the unequally spaced actuator produced a moment of 1.14 Nm. Due to the higher peak moment, the unequally spaced actuator was chosen for further study.

**Figure 6.**
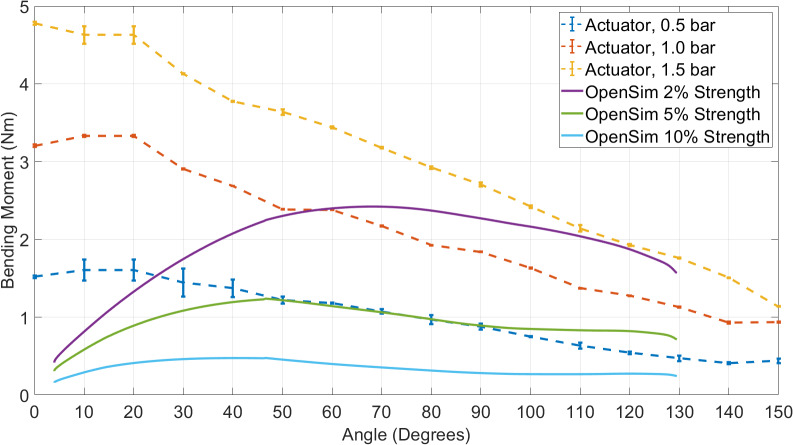
Results of bench testing the unequally spaced actuator, showing actuator moment versus joint angle for 0.5, 1.0, and 1.5 bar internal pressure, over the full range of motion of the joint. The curves of the OpenSim 2%, 5%, and 10% strength requirements are also shown for comparison.

In subsequent testing, the internal pressure of an unequally spaced actuator was increased to 0.5, 1.0, and 1.5 bar to measure the moment produced throughout the full range, which can be seen in Figure 6. This figure also displays the comparison with the requirement from the OpenSim modeling (see Section 3), displaying the 2%, 5%, and 10% strength curves next to the actuator moment. The three actuator moment curves follow a similar profile to each other, showing inverse proportionality between bending moment and angle (a higher bending moment is produced at lower angles). It also shows a similar increase from 0.5 to 1.0 bar as 1.0 to 1.5 bar, suggesting a proportional relationship between pressure and moment. The ability of the actuator to meet the OpenSim requirement at 1.5 bar is shown, with the actuator moment exceeding the requirement throughout the full range of motion.

### 4.4. Attachment method

Effectively transferring the actuator moment to the upper limb while keeping the device easy to don and adapting to different size users required a method of attachment that was different to previously documented designs. As discussed in Section 2, placing the actuator behind the arm significantly limits the range of motion and actuator efficiency. Placing the actuator in parallel with the upper limb allows the actuator to curve naturally and causes no restriction in the flexion of the elbow through its full range of motion (see Figure 1). A potential downside is that it increases shoulder width by 130 mm (distance from the wearer’s skin to the outermost point of the device). To attach the actuator to the limb, low-profile rigid connectors were 3D printed from polylactic acid filament. These transferred the moment produced at the actuator ends to the arm via a TPU strap, secured using Velcro®. The strap around the upper portion of the limb was secured above the bicep (see Figure 1), high enough so that contraction of the biceps muscle during elbow flexion did not cause the strap to become tighter on the arm. The forearm strap was then secured with the elbow fully extended and the actuator at full length. To ensure that the center of the actuator was situated at the elbow joint, adjustment is built into the upper arm attachment to account for different lengths of the limb. The strap width was increased to increase contact area and reduce pressure.

This method of attachment results in a device that causes no active restriction to the movement of the arm when not inflated. The wearable device is also easily donned with assistance and can be sized to fit a wide range of users on either the left or right upper limb. Its low mass (0.295 kg for actuator and attachments) improves wearability. Testing of the device’s ability to transmit the actuator moment to the upper arm is described below.

## 5. Human participant study

### 5.1. Aims

There are three principal aims of the human study. The first aim is to determine the reduction in muscle activity when using the device to perform a prescribed movement. This is a common indicator of the effectiveness of a wearable device, measured using sEMG, and demonstrates the extent to which the device can compensate for reduced muscle strength. Thalman et al., conducted a static test, holding masses of 1.5 and 2.5 kg at 90° elbow flexion. When the device was used for assistance, the reduction in sEMG activity within the bicep was 43% and 63%, respectively. A similar design, known as the “Carry” device (Nassour et al., 2021), also achieved a 50% reduction in sEMG during use. In this study, we aimed to measure the sEMG during both static and dynamic activities.

The second aim is to demonstrate that the wearer is able to perform elbow flexion through the range of motion of the joint, both with and without assistance, without undue discomfort.

The third aim is to measure the percentage of moment transfer from the device to the elbow and compare this to the actuator moment measured during bench testing in Section 4.3. This is important so that the efficiency of the attachment to the body can be understood. Knowing the magnitude of the forces and moments being transferred to the human arm provides an important first step in the characterization of the safety of the device.

### 5.2. Method

A total of 20 participants were recruited, aged 25–74 years, with a mean age of 42 years. The range of height from 157 to 194 cm and weight from 52 to 108 kg, plus the equal split of male and female participants, demonstrates a greater range of participant metrics when compared to the previously cited studies (Koh et al., 2017; Thalman et al., 2018; Nassour et al., 2021; Dávila-Vilchis et al., 2022). Inclusion criteria were “healthy adults without any neurological or musculoskeletal disorders,” and exclusion criteria were “injuries or conditions which restrict the movement of the arm and muscle activation within the arm.” Ethical approval for this study was provided by the Physical Sciences and Engineering Ethics Board of the University of Aberdeen (Application ID 673886). Participants were informed about the study protocol and gave written consent. When fitting the device to participants, we ensured that the strap was sufficiently tight to prevent any translational movement, but without causing discomfort.

**Figure 7.**
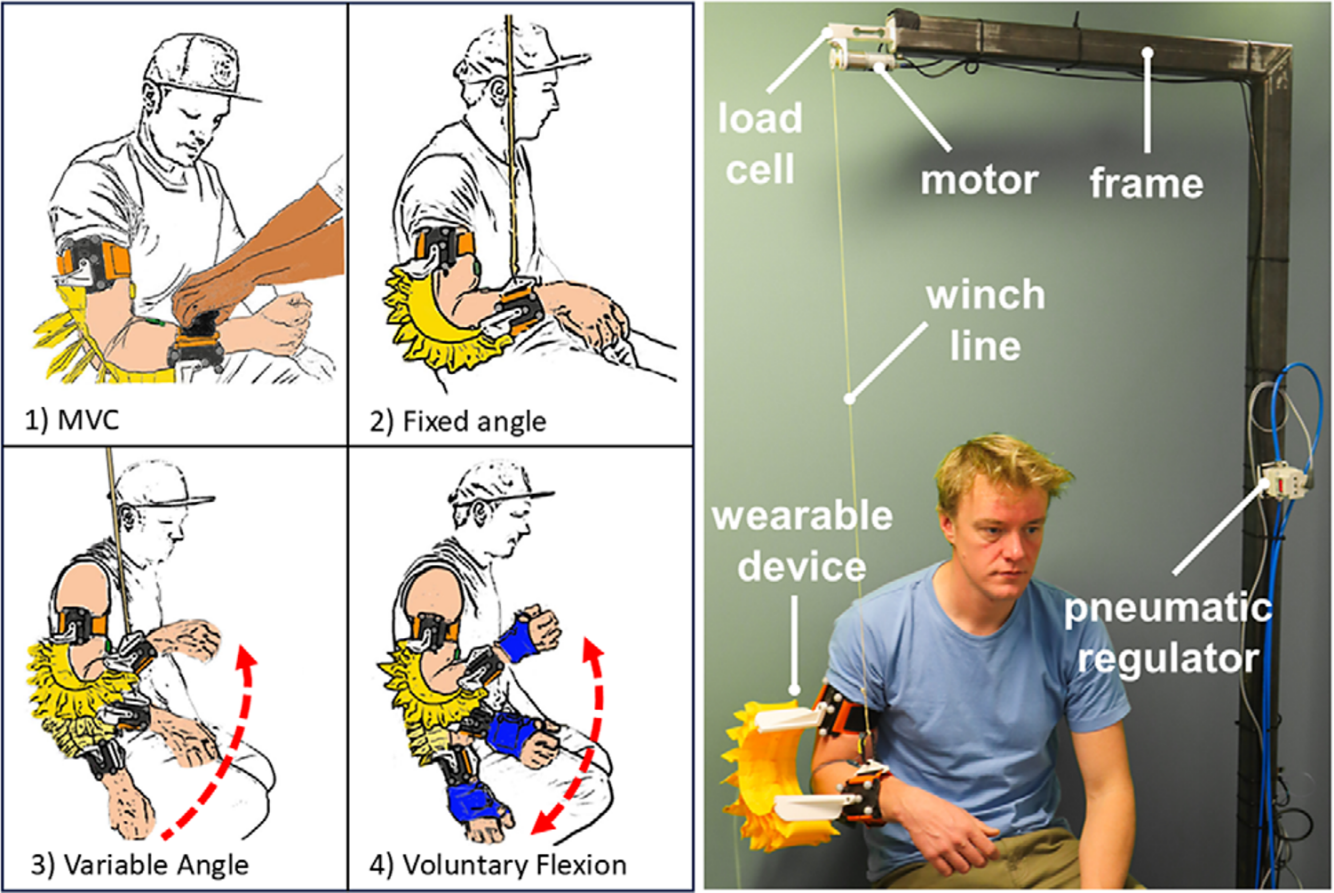
Left, the stages of the human study. (1) Maximum voluntary contraction, (2) fixed angle, (3) variable angle, and (4) voluntary flexion. Right, fixed-angle testing, showing the frame with load cell and motor, with winch line fixed to the forearm attachment while the device is pressurized to measure the moment produced.

To measure the on-body moment, a test apparatus similar to that discussed in McCann et al. (2023) was created (see Figure 7). A 2 m tall frame suspended the load cell (OBUG-20 kg-0.4-000, Applied Measurements) onto which was attached a small 12 V winch motor (950D1001LN, MFA Como Drills). This setup allowed us to measure the weight of an object attached to the winch line. To measure elbow flexion angle, retroreflective marker clusters were incorporated into both the upper and forearm attachment straps (see Figure 1). The position of the marker clusters were tracked by the OptiTrack optical motion capture camera system (Natural Point) and recorded using Motion Monitor (Innovative Sports Training) to record elbow flexion angle.

Preliminary tests showed that a suitable working pressure for the device was 0.5 bar. For lighter participants, values greater than this caused elbow flexion when the limb was required to remain static, meaning it was not possible to determine the moment produced by the device.

For all testing, surface EMG readings were collected from the biceps, triceps, and brachioradialis muscles using a Trigno Quattro 4-channel EMG sensor (Delsys), sampled at 2 kHz. The biceps and brachioradialis were selected as they are key contributors to elbow flexion and can be accessed by surface EMG. The triceps was recorded because it is the main antagonist to this movement, allowing us to check for co-contraction during use. Although the brachialis muscle is also a major contributor to elbow flexion, its activity cannot be measured by surface EMG and so was omitted.

The testing consisted of four stages (see Figure 7).

#### 5.2.1. Maximal voluntary contraction

The first test determined the sEMG reading for a maximal voluntary contraction (MVC) of the target muscles, which was used to scale all subsequent EMG readings. A 20 Hz/450 Hz band-pass filter was applied to remove movement baseline noise, followed by calculations of a moving root-mean-square with a 1 second window (De Luca et al., 2010; Mark Burden et al., 2014). The peak EMG signal for each channel was recorded.

#### 5.2.2. Fixed angle test

This test was used to measure the on-body moment produced by the device, enabling an understanding of the moment transferred to the wearer. During the fixed-angle testing, the participant’s elbow was held at 90°, with the upper arm held vertically against the torso. The hand was kept in a neutral position, with the thumb facing upward. This angle was chosen as the moment arm of the forearm is at its maximum, and the weight is directly opposing the load cell. The actuator pressure was increased from 0 to 0.5 bar as quickly as allowed by the pressure regulator, which took 6–7 seconds. The participants were instructed to relax all muscles in the arm while the internal pressure of the device was increased to a final working pressure of 0.5 bar. By measuring the difference between the initial and final forces on the winch line and the distance from the elbow to the point at which the mass of the forearm and hand are suspended, the moment produced by the device could be calculated. sEMG data were collected to detect any involuntary contraction of the muscles that occurred during the test. A signal below 10% MVC was deemed to be below the threshold for voluntary movement in this test, which has been shown in previous studies to be suitable (Johanson and Radtka, 2006).

#### 5.2.3. Variable angle test

This test aimed to measure the on-body moment through the full range of motion of the elbow. Variable angle testing used the winch to create a flexion movement of the participant’s elbow. The winch line was connected to the forearm attachment of the wearable device, and the winch was powered for a time period controlled using a MATLAB script, via the DAQ (NI-USB-6341, National Instruments), which produced a voltage signal, amplified by an IRF520 MOSFET driver module. The actuator pressure was increased from 0 to 0.5 bar as quickly as allowed by the pressure regulator. The force on the winch line was recorded together with actuator pressure and the angle of the elbow. Assuming negligible angular acceleration throughout the range of motion, the moments around the elbow joint can be expressed by the following equation:(1)

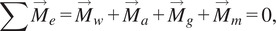



where 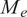 is the moment about the elbow, 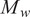 is the elbow moment generated by the winch, 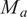 is the actuator moment, 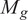 is the moment due to forearm and hand weight, and 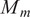 is the moment due to muscle forces. If 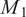 is the moment at zero pressure and 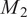 is the moment at 0.5 bar, for each elbow flexion angle:(2)

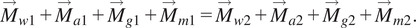

**Figure 8.**
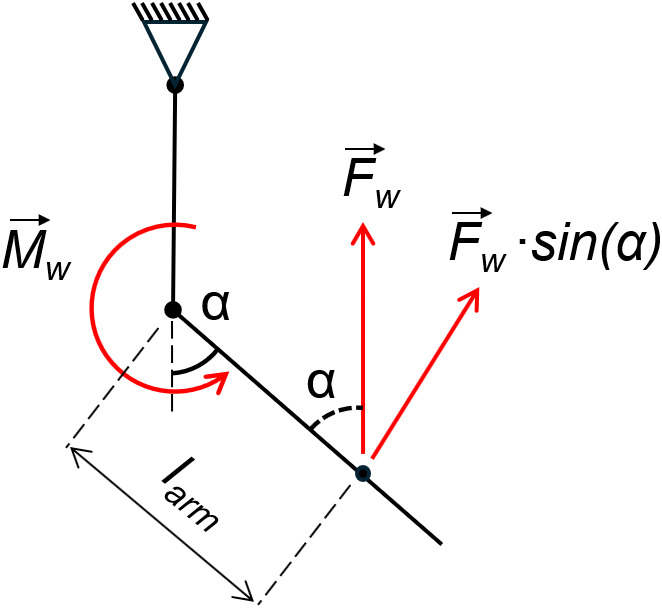
Free body diagram showing the calculation of winch forces on the arm, 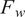 signifying the winch force and 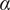 the elbow flexion angle. 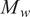 is the resulting winch moment about the elbow.

As participants were asked to relax their muscles (verified by EMG data to ensure no involuntary muscle activity occurred), 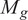 and 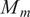 can be assumed to be unchanged with and without the device. At zero pressure, 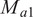 is zero. Therefore, from Equation 2:(3)

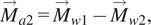



where 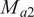 is the applied actuator moment and 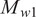 and 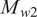 are the winch moments with 0 and 0.5 bar actuator pressure, respectively. The moment contribution from the winch can be calculated from the load cell’s force measurement. The load cell measured the vertical component of the winch line tension; therefore, the winch moment applied around the elbow joint was calculated as follows:(4)

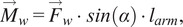



where 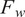 is the measured winch force, 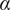 is the elbow flexion angle, and 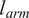 is the distance from the elbow to the forearm attachment (see Figure 8). By comparing the results for 0 and 0.5 bar actuator pressure, the moment contribution throughout the whole range of motion can be calculated.

#### 5.2.4. Voluntary flexion test

This test was used to determine the maximum range of motion of the device and the reduction in muscular activity from wearing the device for a prescribed movement. During the voluntary flexion testing, participants performed the prescribed movement both with and without the assistance of the device, while also using additional weights of 1 and 2 kg added to their hand. The required movement was full flexion and extension of the elbow, repeated five times for each combination of device assistance and weight. To help keep the speed of movements identical, the participant was instructed to track the movement shown in a video recording. The actuator pressure was increased from 0 to 0.5 bar as quickly as allowed by the pressure regulator. EMG and elbow flexion angle data were collected to determine the muscle activation throughout all movements. Peak EMG was identified for each flexion movement, and the EMG curve was integrated for the biceps and brachioradialis muscles (which contribute to elbow flexion) over the periods associated with elbow flexion. The voluntary effort could be compared between the six different scenarios (0, 1, and 2 kg additional hand mass, conducted with and without assistance from the device). The triceps EMG is plotted to check that no significant co-contractions occurred during elbow flexion.

### 5.3. Results

#### 5.3.1. Maximal voluntary contraction

The maximum flexion moment generated by the participants ranged from 19 to 55 Nm with a mean of 33 Nm (SD = 16 Nm).

#### 5.3.2. Fixed-angle test

During the fixed-angle test, the mean moment generated was 0.94 (SD = 0.24) Nm. The moment arm varied between participants due to their different arm lengths, with a mean of 178 mm (SD = 13). When compared with the results of the bench testing of the actuator, this corresponds to 82% of the moment produced by the actuator being transferred to the wearer at a 90° angle. The most probable cause of this loss is the difference between the fully rigid connections of the bench test and the nonideal connections through the soft tissue of the human participants.

#### 5.3.3. Variable angle test

**Figure 9.**
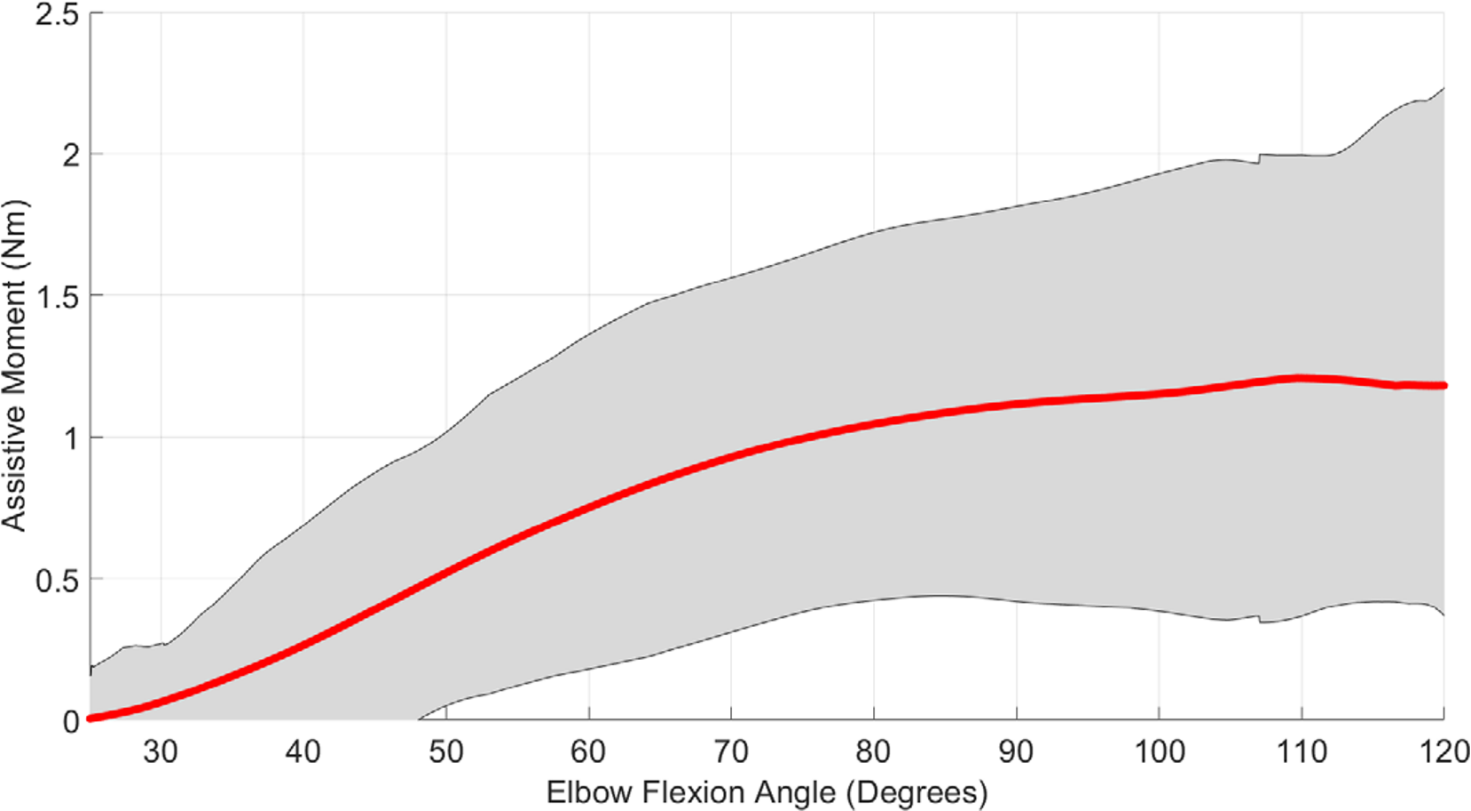
Assistive moment was measured during the variable angle testing at increasing elbow flexion angles for all participants. The mean moment is shown in red, with the full range of measured values depicted by the grey shaded region.

The moment generated by the device during the variable angle testing can be seen in Figure 9, which plots the moment against the elbow flexion angle for all participants. The red line depicts the mean result, with the grey area depicting the upper and lower bounds for all participants. The peak mean bending moment of 1.2 Nm occurred at 110° of elbow flexion. The wide range of these results, whereby the moment for different users varies by up to 100% of the mean, shows the variability between different users of the device.

#### 5.3.4. Voluntary flexion test

The mean range of motion of the participants during the voluntary flexion test was 127° (SD = 12) without assistance and 132° (SD = 13) when assistance was provided. The mean peak elbow flexion angle was 149°, showing that the range of motion used was at the peak flexion range and that participants were not fully extending their arm. This is a typical pattern of use, as patients were not instructed to actively extend their elbows.

**Figure 10.**
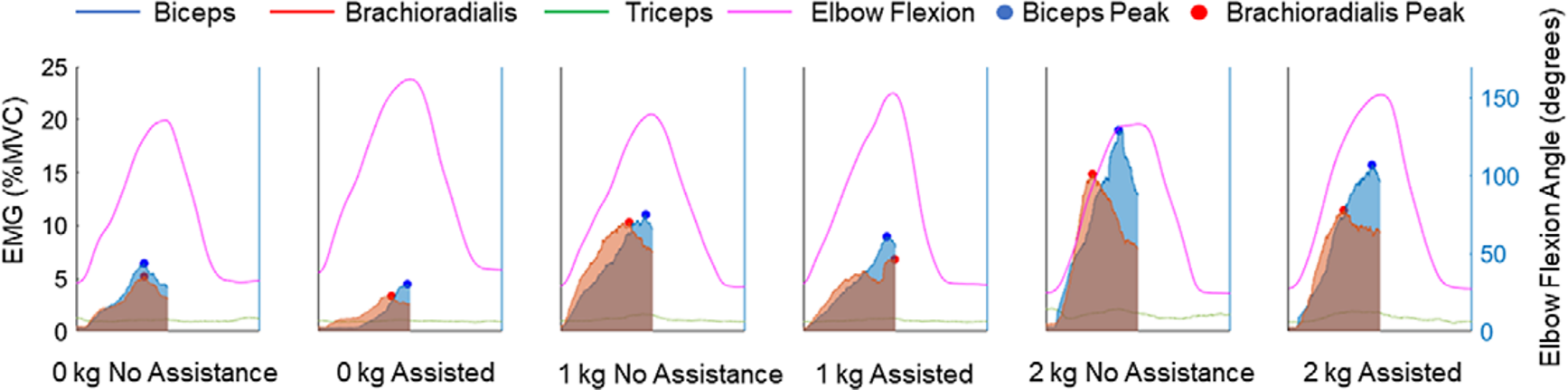
A sample sEMG plot from the flexion test of a single participant. This shows %MVC for each muscle for one of five repetitions of an elbow flexion-extension movement, along with the elbow flexion angle for reference. The shaded regions represent the integrated portion relating to the flexion part of the movement, where assistance can be given, and the dots represent the peak sEMG for each flexion movement.

Figure 10 shows the EMG recordings for the biceps, triceps, and brachioradialis for a sample participant during the voluntary flexion testing. The shaded regions represent the integrated signals measured during elbow flexion, and the colored dots represent the peak readings for each movement. The elbow flexion angle is plotted to check the time intervals of the flexion movement. The increased magnitude of EMG readings as the mass in the hand increases can be clearly seen, as well as the decreased magnitude between unassisted and assisted movements. The sum of the peak and integrated sEMG signals were calculated for each test and the reduction in sEMG calculated between the assisted and unassisted movements as a percentage of the unassisted result.

**Figure 11.**
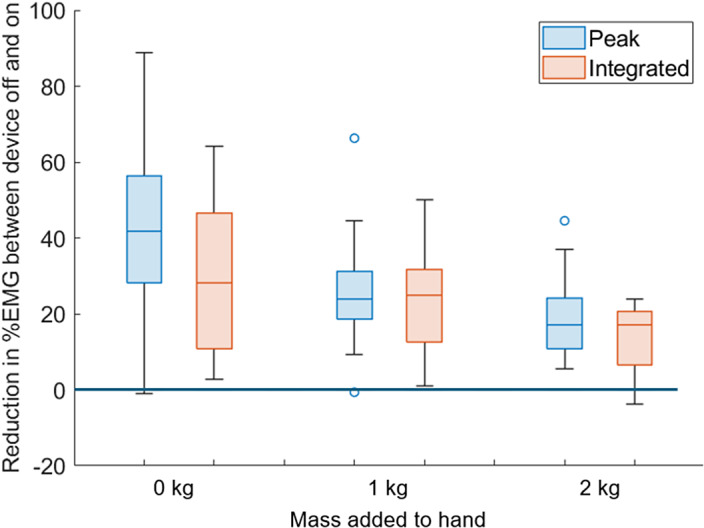
Percentage reduction of integrated and peak sEMG when using the wearable device. Values shown are for all participants with 0, 1, and 2 kg additional mass added to the hand.

Figure 11 shows the sEMG results for all participants, giving the percentage reduction in measured sEMG when using the wearable device versus completing the task unaided, for the 0, 1, and 2 kg additional masses added to the hand. The median (IQR) peak sEMG reductions were 41.7% (28.1–56.4), 24.0% (18.5–31.2), and 16.5% (12.4–21.6) for the 0, 1, and 2 kg loading conditions, respectively. The median (IQR) integrated EMG signal reductions were 27.8% (10.7–46.5), 24.8% (12.6–31.6), and 17.2% (10.8–20.5), respectively. This shows a 39% (integrated sEMG) to 59% (peak sEMG) reduction in the level of assistance for the increased mass in the hand. This is expected, as the constant moment produced by the device provides less relative assistance as the moment required to complete the movement increases. The absolute reduction in %MVC was also calculated, with reductions of 3.6% (1.2–10.2), 6.1% (1.5–9.4), and 6.1% (2.8–9.9) for the 0, 1, and 2 kg additional mass conditions. A two-way ANOVA test checked the statistical significance of both the peak and integrated results. This showed strong statistical significance that the device reduced measured sEMG when assistance was provided (*p* < 0.001), but that the reduction in sEMG between the three weight categories was not significantly different (peak sEMG, *p* = 0.02; and integrated sEMG, *p* = 0.009). This shows that the device can effectively assist users by reducing the muscular effort required to perform an elbow flexion movement.

## 6. Discussion and further work

The study gives us an insight into the potential benefits of coupling biomechanical modeling with wearable device design. The experiments conducted show the performance of the decoupled actuator design and afford a quantitative analysis of physical human–robot interaction.

### 6.1. Biomechanical modeling

The modeling described in Section 3 defined the basic device requirements. The use of the MoBl-ARMS model only allows for an understanding of the requirements of an average male user, and further study would be required to understand how these results would change for subjects with different anthropometry or muscle strength. The restricted elbow flexion range of the model was also a limiting factor, allowing analyses only up to 130°, as opposed to the full 150° of the average human elbow.

Further work in this area would incorporate the device as a model with geometrical and inertial properties. This would more accurately model the interaction between the device and the human, and also include such factors as device weight and inertia, to optimize the geometry and potentially provide insight into how to fit the device to each individual.

### 6.2. Device development

When compared to other pneumatic exosuit designs (Koh et al., 2017; Dávila-Vilchis et al., 2022), our device has a higher mean range of motion, 132 (SD = 13)°, with the highest of other pneumatic exosuit designs achieving only 118.9° (Koh et al., 2017). In addition, our device achieves a mean peak flexion of 149° (SD = 8.5), which is very close to the maximum peak flexion of 150° and higher than the 118.9° reported for existing devices (Koh et al., 2017). While other wearable devices show a higher bending moment, such as in the work by Thalman et al. (2018) and Dávila-Vilchis et al. (2022), the range of motion of these devices was very low, at 107° and 70°, respectively, which would limit the capacity to perform rehabilitation exercises aimed at restoring ADL capabilities.

While this study has focused on assisting elbow flexion, the opposing movement of elbow extension is also necessary to conduct ADLs, as well as accurately control elbow position and stiffness. It has been shown that a simple straight pneumatic chamber can be used to effectively assist elbow extension (Nam, 2022) and including such an actuator into a future prototype would allow this antagonistic motion.

The device was designed with the goal of having one model fit all users, and this study has shown that it is possible for a wide range of users to wear it effectively, as demonstrated in the results of the voluntary flexion test (see Figure 11).

By modeling the geometry of the actuator in Section 4.2, it was possible to connect the effect of changes to the spacing and height of the individual chambers to the curvature of the actuator. Further study into the interaction between these chambers and the internal pressure could allow further characterization of the bending moment produced by the actuator. The characterization shown in Section 4.3 shows that increases in pressure lead to an increased bending moment, with a 430% increase in pressure producing a 370% increase in moment. The need for high-pressure air and high flow rates requires larger and less portable compressors. However, in recent years, portable pneumatic actuation units capable of driving these robots have become available (Sridar et al., 2020; Joshi, 2021).

Control of the device was achieved by manually modifying pressure. Potential future work would include developing a closed-loop system where the device pressure could be automatically adjusted based on the user’s desired elbow flexion angle.

### 6.3. Human study

The aims of the human study were to determine the effectiveness of the device by showing the reduction in muscle activity from using the device, demonstrating that the wearer is able to use the device within a wide range of movement of the joint, and to measure the percentage of moment transferred to the user. This healthy participant testing is a necessary prerequisite before future studies involving the clinical populations that require upper-limb rehabilitation can be performed.

The voluntary flexion test results show that the mean flexion range was 88% of the theoretical maximum and that the majority of participants reached the peak elbow flexion angle of 150°, suggesting that restrictions occurred on the fully extended end of the range. As participants were not instructed to fully extend their elbows during trials (which requires contraction of the triceps), this does not signify a restriction in range of motion from the device.

The median peak sEMG reduction was 41.7% with zero additional hand weight. Comparing these results to those of other studies is not simple, as the protocols varied significantly, typically using static holding tests and with devices operating at higher pressures. However, the results reported show similar levels of reductions ranging from 43% to 63% (Thalman et al., 2018), and 35% (Nassour et al., 2021). This shows that the level of assistance provided by our device is in line with existing devices.

The median integrated sEMG reduction was 27.8% with additional hand weight, showing that the overall reduction throughout the movement is lower than the reduction in peak sEMG. However, this shows that the reduction in muscle activity is sustained throughout the movement, demonstrating the continuous assistance provided. None of the above studies compares peak and integrated sEMG.

The static and variable angle testing results inform the percentage of moment transferred to the user. The mean static moment at 90° was 0.94 (SD = 0.24) Nm, which is 82% of the bench test result. This test was conducted at 90°, the angle where maximum elbow flexion moment occurs. However, future tests could compare this result over a range of elbow flexion angles. These tests could be further enhanced in the future by considering the impedance of the human arm and that of the mannequin.

## 7. Conclusions

This article demonstrates that our decoupled soft wearable robot can assist with elbow flexion throughout the entire range of motion of elbow joint (up to 149°, SD = 8.5). At 1.5 bar, the actuator was able to generate a moment that exceeds the requirement of all simulated users in our biomechanical model, as shown in Figure 6. To show the effectiveness of the device in assisting human movement, we performed a series of tests with healthy human subjects. We found significant sEMG reductions of the elbow flexors during controlled exercises, indicating effective moment transfer to the user. We showed that there is a decreasing level of assistance for the increased mass in the hand, which translates to objects was held in the hand by the user.

The human participant tests also showed that 82% of the moment produced by the actuator being transferred to the wearer at 90°, which is significantly higher than that shown for the only known comparable test, completed for the shoulder (McCann et al., 2023). No direct comparison was possible, as this study provides the first quantitative evaluation of the moment transfer from the device to the user’s elbow, paving the way for other studies to follow. This study also showed that the proposed decoupled device design can support elbow flexion in healthy users with varying body types, although further research is needed to thoroughly characterize this aspect. The variance in moment required by different users highlights the need for user-adaptive control, which is a common requirement for most wearable devices.

This study introduces a lightweight and low-cost wearable device that avoids restricting the range of motion and characterizes physical human–robot interaction in a quantitative manner. These requirements are a crucial step toward the development and evaluation of an effective soft robotic device for rehabilitation.

## Data Availability

The data that support the findings of this study are available from the corresponding author, MEG, upon reasonable request.
